# AI exposure, occupational mobility, and post-transition job quality in China: labor-saving and labor-augmenting channels

**DOI:** 10.3389/fpsyg.2026.1799093

**Published:** 2026-07-28

**Authors:** Ling Zhang, Zhenzhen Liu

**Affiliations:** School of Economics, Jinan University, Guangzhou, China

**Keywords:** artificial intelligence, career adaptation, China, labor-augmenting exposure, labor-saving exposure, occupational mobility, post-transition job conditions

## Abstract

This study examines how exposure to artificial intelligence (AI) shapes Chinese workers’ occupational mobility and how the consequences differ across AI’s labor-saving and labor-augmenting channels. Using 2,446 worker-year observations from the China Labor Force Dynamics Survey 2012–2018, we measure occupation-level AI exposure as the semantic similarity between Chinese task descriptions and AI patent texts and decompose it along routine and non-routine task lines. Causal identification uses an instrumental variable that interacts 2010 baseline occupation-level exposure with lagged cumulative U.S. patent stocks from the USPTO AI Patent Dataset. The 2SLS estimates yield three findings. First, a one-log-unit increase in total AI exposure raises minor-group switching by 3.6 percentage points and intermediate-group switching by 3.4 points, with no significant effect at the major-group level. In the decomposed estimates, only the labor-augmenting component has significant positive coefficients beyond the minor-group margin, reaching 4.6 points at the intermediate and 6.9 points at the major-group distance. The labor-saving coefficients are statistically indistinguishable from zero in the full-sample switching-incidence IV estimates. The short-distance response is concentrated among women, and among less-educated workers labor-saving exposure predicts lower and labor-augmenting exposure higher major-group mobility, suggesting a labor-saving-related barrier to long-distance transitions. Second, among switchers, labor-augmenting exposure exhibits positive but attenuated origin–destination sorting in all three switching samples. Labor-saving sorting is positive in the minor-group sample, statistically indistinguishable from zero in the intermediate-group sample, and negative in the major-group sample. Third, among switchers, greater labor-saving exposure in the origin occupation is associated with longer hours in the minor- and intermediate-group samples and with lower skill-match satisfaction in all three samples, whereas favorable associations with origin labor-augmenting exposure are confined to specific samples. The study extends organizational-psychology research by linking instrumented AI exposure to occupational switching as an observable career-adaptation behavior and to the fit and demand conditions of destination jobs. The findings suggest targeted reskilling and organizational support for less-educated workers in occupations with high labor-saving exposure.

## 1 Introduction

Since the mid-2010s, artificial intelligence (AI) has diffused rapidly through machine learning, natural language processing, and generative models, reshaping the boundaries of work tasks. Unlike earlier automation that mainly replaced manual routine work, AI can directly participate in cognitive activities such as information processing, text generation, and data analysis. AI thus operates on labor through two opposing channels: a labor-saving channel that exerts displacement pressure on workers whose tasks are highly automatable, and a labor-augmenting channel that generates productivity-enhancing complementarities for workers whose tasks AI augments (Acemoglu and Restrepo, 2018, 2019). Understanding how workers respond is central to both labor economics and organizational psychology, because occupational mobility is one of the most consequential career-adaptation behaviors workers undertake when technology reshapes their job content (Savickas, 2013; Spurk et al., 2019). Whether these transitions represent defensive responses to displacement or strategic moves toward complementary opportunities matters for the fit and demand conditions of the jobs workers enter. Perceived skill match and quantitative job demands are both relevant to subsequent well-being and engagement.

The existing AI-and-labor literature has converged on three stylized facts. First, AI does not appear to generate large aggregate-employment declines: U.S. firm-level evidence shows that AI-adopting firms grow faster (Babina et al., 2024), and 23-country cross-country evidence on AI exposure finds no systematic link with employment declines (Georgieff and Hyee, 2022). Evidence on the broader automation literature is mixed: significant negative effects on prime-age labor-force participation across 23 advanced economies (Grigoli et al., 2020) coexist with benign aggregate effects from U.S. patent-based automation measures (Mann and Püttmann, 2023). Occupational-level routine-task displacement estimates remain contested across countries and methodologies, partly reflecting differences in measurement strategy and identifying variation.

Second, micro-level evidence shows AI reshapes task bundles unevenly across skill groups: in audit firms, AI adoption concentrates labor demand and reduces employment of junior auditors (Fedyk et al., 2022); within occupations more broadly, AI raises the skill frontier (Braxton and Taska, 2023; Deming and Noray, 2020); and generative AI hiring exhibits a seniority bias (Hosseini Maasoum and Lichtinger, 2025). In contrast to the firm-level evidence of net employment growth (Babina et al., 2024), micro-evidence on routine-task workers documents substantial downgrading (Cortes, 2016; Feigenbaum and Gross, 2024).

Third, recent work distinguishes the automation-oriented and augmentation-oriented components of AI. Autor et al. (2024) link automation-oriented patents to reduced labor demand and augmentation-oriented patents to new-task creation; Eloundou et al. (2024) and Brynjolfsson et al. (2025) provide direct evidence on the labor-augmenting effect of generative AI on less-experienced workers. Null-result evidence (Humlum and Vestergaard, 2025) further shows that the estimated labor-market impact of AI is sensitive to setting and method.

Credible measurement of occupational AI exposure is central to this literature. Building on the task-based tradition (Autor et al., 2003; Autor and Dorn, 2013; Frey and Osborne, 2017), patent-text-based approaches link AI capabilities to occupational tasks (Webb, 2020; Felten et al., 2018, 2021). Kogan et al. (2023) introduce the labor-saving versus labor-augmenting distinction, enabling separation of the two channels. Despite this progress, three gaps remain. First, most occupational AI exposure indices remain one-dimensional, conflating labor-saving and labor-augmenting forces. Second, micro evidence on occupational mobility rarely organizes switching by transition distance to distinguish short-distance re-matching within nearby occupations from moves across broad occupational groups. Third, for China, studies that combine locally grounded task texts with longitudinal worker microdata to construct and validate decomposed exposure measures remain scarce, and the evidence base on AI-related reallocation patterns and post-transition conditions is correspondingly limited.

This study addresses these gaps. We develop a task-text-based measure of occupational AI exposure for China, decomposed into labor-saving and labor-augmenting components. Three margins are examined in sequence: the incidence of switching, destination sorting among switchers, and post-transition job conditions. The empirical analysis uses individual data from the China Labor Force Dynamics Survey (CLDS) 2012–2018, the 2015 *Chinese Standard Classification of Occupations* for task content, and the patyee database for Chinese AI patents. Causal identification uses an instrumental variable that interacts 2010 baseline occupation-level exposure with lagged cumulative U.S. patent stocks from the U.S. Patent and Trademark Office (USPTO) Artificial Intelligence Patent Dataset (AIPD), supplemented by propensity-score matching.

Three findings emerge across the three margins. First, AI exposure increases short-distance switching, while the labor-augmenting component exhibits a positive gradient across transition distances. Second, among switchers, labor-augmenting exposure exhibits positive but attenuated origin–destination sorting in all three switching samples, whereas labor-saving sorting is positive in the minor-group sample, statistically indistinguishable from zero in the intermediate-group sample, and negative in the major-group sample. Third, among switchers, greater labor-saving exposure in the origin occupation is associated with longer hours in the minor- and intermediate-group samples and with lower skill-match satisfaction in all three samples, whereas favorable associations with origin labor-augmenting exposure are confined to specific samples.

The study makes three contributions. First, it develops a Chinese-context task-based AI exposure measure with explicit decomposition into labor-saving and labor-augmenting components; existing one-dimensional measures mask channels with contrasting mobility effects and qualitatively different post-transition implications. Second, it organizes the empirical analysis along an explicit transition-distance dimension and a three-layer chain from switching incidence to destination sorting and post-transition outcomes, making the destination-job conditions associated with AI-related mobility directly visible. Third, the study contributes to organizational psychology by examining occupational mobility as an observable career-adaptation behavior under technological change. Complementing research that treats career adaptability primarily as an individual resource, we link instrumented AI exposure to workers’ mobility behavior and to the fit and demand conditions of their destination jobs.

The remainder of the paper is organized as follows. Section 2 develops the theoretical framework and states the hypotheses. Section 3 describes the data and specifies the identification strategy. Section 4 presents the baseline, instrumental-variable, and supporting empirical results. Section 5 discusses the findings. Section 6 concludes.

## 2 Theoretical framework

We motivate the empirical analysis with a task-based conceptual framework drawn from Acemoglu and Restrepo (2018, 2019), and Kogan et al. (2023). Each occupation o at time t comprises a bundle of tasks partitioned into routine tasks R and non-routine tasks N , following the canonical task-content classification of Autor et al. (2003) and Acemoglu and Autor (2011). Routine tasks are codifiable, repeatable, and executable by software or machine; non-routine tasks require creative thinking, complex judgment, interpersonal interaction, or higher-order cognitive processing. Workers in occupation o are exposed to AI along two distinct dimensions. The first is labor-saving exposure $X_{ot}^{R}$ , which captures the extent to which AI can substitute for tasks currently performed by labor in o . The second is labor-augmenting exposure $X_{ot}^{N}$ , which captures the extent to which AI can complement workers in performing non-routine tasks. These two dimensions correspond to the displacement and productivity effects in Acemoglu and Restrepo (2018, 2019): labor-saving exposure captures the substitutability of routine tasks, whereas labor-augmenting exposure captures complementarity with existing non-routine tasks. A third possibility, the reinstatement effect through which technology creates new task categories, is conceptually related to labor demand but is not directly observed by our task-similarity measure; we therefore leave it for future research. This distinction maps directly onto the measurement strategy in Section 3.2, which adapts Kogan et al.'s (2023) patent-text approach to Chinese occupational task descriptions.

The labor-augmenting channel can affect mobility through more than in-place productivity gains. If AI complementarity only raises productivity in the origin occupation, it may increase the value of staying rather than switching. A positive distance gradient therefore requires a second, more tentative pathway: AI augmentation may expand the set of cognitively demanding destinations that workers from less cognitively intensive origins can plausibly access. We refer to this candidate pathway as complementary upgrading. It differs from a portable-skill interpretation, in which long-distance mobility reflects non-routine skills already accumulated in the origin occupation and transferable across jobs. Because we do not observe worker-level AI adoption, skill acquisition, or learning, we treat complementary upgrading as a candidate interpretation rather than a demonstrated mechanism; worker selection and pre-existing human capital remain alternative explanations.

Workers face a discrete switching decision: stay in occupation o or move to destination occupation $o^{\prime }$ . The net value of switching depends on the difference between the value of the outside option $V_{io^{\prime }t}$ and the value of staying $V_{iot}$ , minus a switching cost $c\left(d\left(o,o^{\prime }\right)\right)$ that increases with occupational distance and reflects the loss of occupation-specific human capital (Kambourov and Manovskii, 2009). Labor-saving exposure is expected to lower the value of staying through displacement pressure and therefore to raise switching incentives, especially at shorter distances where switching costs are smaller. Labor-augmenting exposure has an ambiguous effect on mobility: the in-place productivity channel raises the value of staying, whereas the candidate complementary-upgrading pathway may raise the value of more distant destinations with greater cognitive-task content. Whether the net labor-augmenting effect on switching is positive, and at which distances, therefore depends on the balance between these channels and the magnitude of switching costs.

From an organizational-psychology perspective, the dual-channel structure maps onto the distinction between defensive and exploratory career-adaptation behavior (Savickas, 2013; Spurk et al., 2019). Labor-saving exposure is consistent with defensive mobility toward lower-risk or skill-adjacent destinations, whereas labor-augmenting exposure, if the candidate complementary-upgrading pathway dominates, is consistent with exploratory mobility toward more cognitively demanding work. We distinguish career adaptability as a psychosocial resource from career-adaptation behavior as an observable response; the CLDS measures the latter, so the education heterogeneity in Section 4.3 is interpreted as human-capital stratification rather than as a direct test of adaptability resources. Beyond the switching decision itself, we treat destination-job person–job fit and job demands as organizational-psychology outcomes of mobility, constructs that the Job Demands–Resources framework links to strain and engagement (Bakker and Demerouti, 2017). Their channel-specific prediction is stated in Hypothesis H3 below, and their CLDS operationalization is described in Sections 3 and 4.4.2.

This framework yields three testable hypotheses that we examine in the empirical specifications of Sections 3.3 and 4.

*Hypothesis H1.* Higher labor-saving exposure is expected to raise the probability of switching through displacement pressure on the value of staying; because switching costs rise with occupational distance, this effect should be strongest at shorter transition distances. Labor-augmenting exposure has a theoretically ambiguous net effect: the in-place productivity channel raises the value of staying, whereas the candidate complementary-upgrading pathway may raise the value of more distant outside options when AI augmentation expands the accessible cognitive frontier. If the complementary-upgrading pathway dominates, the labor-augmenting effect should increase with transition distance, in contrast to the cost-driven distance gradient expected for labor-saving exposure.

*Hypothesis H2.* Conditional on switching, a defensive-sorting account predicts that workers from more labor-saving-exposed origins enter destinations with lower labor-saving exposure than workers from less-exposed origins. On the labor-augmenting dimension, a portable-skill mechanism predicts positive origin–destination sorting, whereas the candidate complementary-upgrading pathway does not require such positive sorting.

*Hypothesis H3.* Conditional on switching, greater labor-saving exposure in the origin occupation is expected to predict less favorable post-transition job conditions, whereas greater origin-occupation labor-augmenting exposure is expected to predict more favorable ones. We proxy perceived person–job fit by self-reported skill-match satisfaction and job demands by weekly working hours: H3 predicts that labor-saving-exposed switchers enter destinations with worse skill match and longer hours, while labor-augmenting-exposed switchers enter destinations with better skill match and shorter hours.

## 3 Materials and methods

### 3.1 Data sources

*Microdata.* The microdata source is the China Labor Force Dynamics Survey (CLDS), administered by the Center for Social Survey at Sun Yat-sen University. We restrict the sample to individuals aged 16–65 who report valid five-digit occupation codes for both the current and the previous job. After excluding missing values, the final estimation sample comprises 2,446 worker-year observations. City-level covariates are drawn from the *China Urban Statistical Yearbook*.

*Occupational task texts.* Task descriptions are extracted from the 2015 edition of the *Chinese Standard Classification of Occupations*. We extract 9,334 task statements covering all unit groups at the five-digit level. Each task statement is classified as *routine* or *non-routine* using GPT-3.5 with the task-content criteria of Autor et al. (2003) and Acemoglu and Autor (2011) stated in Section 2. The labeling yields 5,839 routine task statements (62.6%) and 3,495 non-routine task statements (37.4%), broadly consistent with the 62% routine share that Kogan et al. (2023) report for the U. S. Dictionary of Occupational Titles (see Supplementary material Section A.2 for human and cross-model validation).

**Figure 1:**
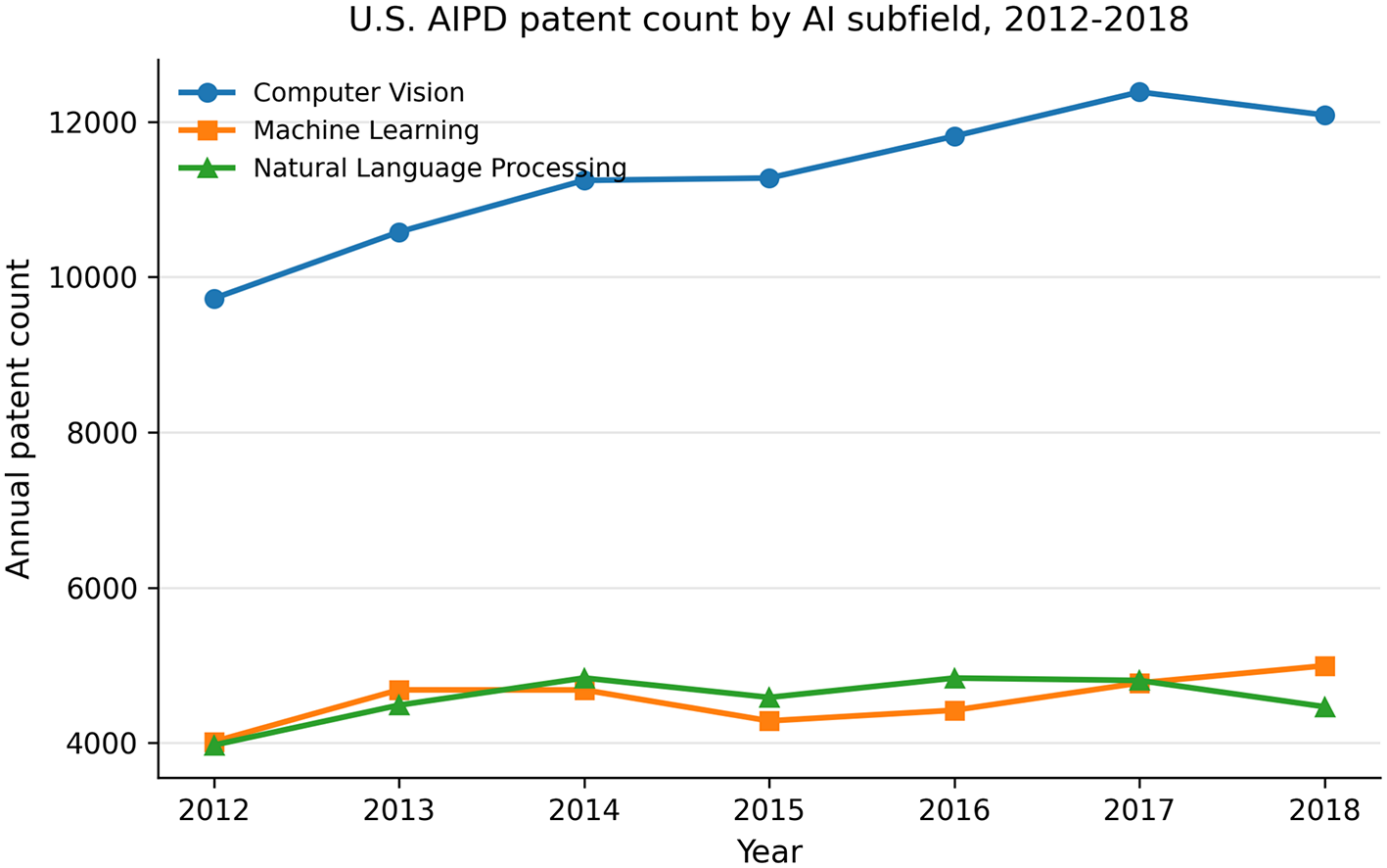
U.S. AIPD patent count by AI subfield, 2012–2018. Annual count of U.S. AI patents in the three subfields used to construct the instrument: computer vision, machine learning, and natural language processing. The displayed range matches the CLDS sample period; the IV uses one-year-lagged cumulative stocks of these three subfields. Source: USPTO AIPD (Giczy et al., 2022).

*AI patent texts.* Chinese AI patent texts come from the patyee database. We retrieve all 2010–2018 patents whose title or abstract contains at least one of 93 AI-related keywords spanning AI hardware, core methods such as machine learning, deep learning, and neural networks, and major application domains (see Supplementary material Section C for the full keyword list). For each retrieved patent, we concatenate the title and abstract. For the instrumental variable specified in Section 3.3, we additionally use the AIPD, publicly released by the USPTO’s Office of the Chief Economist; the dataset construction is described in Giczy et al. (2022). We use the three subfields with the broadest task coverage and most direct mapping to occupational labor inputs: machine learning, computer vision, and natural language processing. Figure 1 reports annual U.S. AIPD patent counts in these three subfields, which enter the IV in Section 3.3 as one-year-lagged cumulative stocks.

### 3.2 Construction of key variables

*Dependent variables: occupational switching.* We define three indicators of occupational switching based on the nested structure of the five-digit Chinese occupational code: a *minor-group switch* indicates a change at any of the five digits, an *intermediate-group switch* indicates a change at the first three digits, and a *major-group switch* indicates a change at the leading one-digit class. The three indicators are nested by construction: a major-group switch implies an intermediate- and a minor-group switch.

*Key explanatory variable: AI exposure.* We construct occupation-level AI exposure as the semantic similarity between patent texts and occupational task descriptions, broadly following the approach of Kogan et al. (2023) but with three implementation choices appropriate to the Chinese-language corpus: a multilingual sentence-transformer encoder; FAISS nearest-neighbor retrieval; and a sparsification threshold re-calibrated to the Chinese-language similarity distribution. The construction proceeds in three steps; the full algorithm and intermediate equations are in Supplementary material Section A. Task statements and patent texts are encoded into 384-dimensional dense vectors using the paraphrase-multilingual-MiniLM-L12-v2 sentence-transformer model with $L_{2}$ normalization, so that inner products equal cosine similarities. For each year t , the FAISS library (Johnson et al., 2021) retrieves the top K=50 most similar patents for each task statement; similarity scores are sparsified at the 60th-percentile threshold of the Chinese task–patent similarity distribution, and the truncated similarities are summed to yield a task-level AI relatedness score. Occupation-level exposure measures are then constructed by summing task-level scores over all routine, all non-routine, and the union of task statements within each occupation o :


$$\xi_{o,t}^{c}=\underset{i\in c}{\sum }x_{i,t},\;\;c\in \left\{R,N\right\}$$ (1)

where R and N index the routine and non-routine task subsets, respectively, within occupation o . The total exposure is defined as $\xi_{o,t}^{\mathrm{total}}=\xi_{o,t}^{R}+\xi_{o,t}^{N}$ . Because the raw exposure measures are right-skewed, we apply the transformation ln(1+ξ) before estimation; the resulting distributions of the three exposure measures across the 190 five-digit occupations in the estimation sample are shown in Supplementary Figure A1.

The two key construction parameters, the retrieval cap K and the 60th-percentile sparsification threshold, are justified in Supplementary material Section A.1 and stress-tested in Supplementary material Section A.4. The sensitivity analysis re-estimates the main specification (the instrumental-variable model of Section 3.3) over a one-at-a-time grid of (K ∈ {30, 50, 70} and *τ* ∈ {0.50, 0.60, 0.70, 0.80}), together with the GLM-4.6-labelled variant introduced in Section A.2; all alternative estimates fall within the baseline 95% confidence interval.

*Interpretive boundary of the exposure index.* The exposure measure in Equation 1 captures the potential applicability of AI to occupational tasks, not realized workplace adoption; patent disclosure typically precedes industrial diffusion by several years. The index should therefore be read as a frontier-exposure measure with task-level resolution rather than a record of direct firm-level AI use. The direction of any resulting bias is not known; the instrumental-variable strategy in Section 3.3 addresses some forms of measurement error in the exposure index only under the maintained relevance and exclusion assumptions.

### 3.3 Empirical specification

*Baseline Probit model.* To estimate the relationship between AI exposure and the probability of occupational switching, we fit the following Probit model:


$$\mathrm{Pr}\left({\mathrm{Switch}}_{it}=1\right)=\Phi \left(\beta_{0}+\beta_{1}{\mathrm{AI}}_{jt}+X_{it}^{\prime }\gamma +W_{it}^{\prime }\delta +\lambda_{t}+\mu_{p}\right)$$ (2)

where ${\mathrm{Switch}}_{it}$ is one of the three occupational-switching indicators; ${\mathrm{AI}}_{jt}=\ln\left(1+\xi_{jt}\right)$ is the log AI exposure of origin occupation j in year t ; t is the reported end year of the previous job for newly sampled respondents and the preceding survey-wave year for panel respondents, for whom exact transition timing is unavailable; $X_{it}$ is a vector of individual covariates; $W_{it}$ is city-level covariates; $\lambda_{t}$ and $\mu_{p}$ are year and province fixed effects. Standard errors are clustered at the origin-occupation level. We report average marginal effects (AMEs).

**Figure 2:**
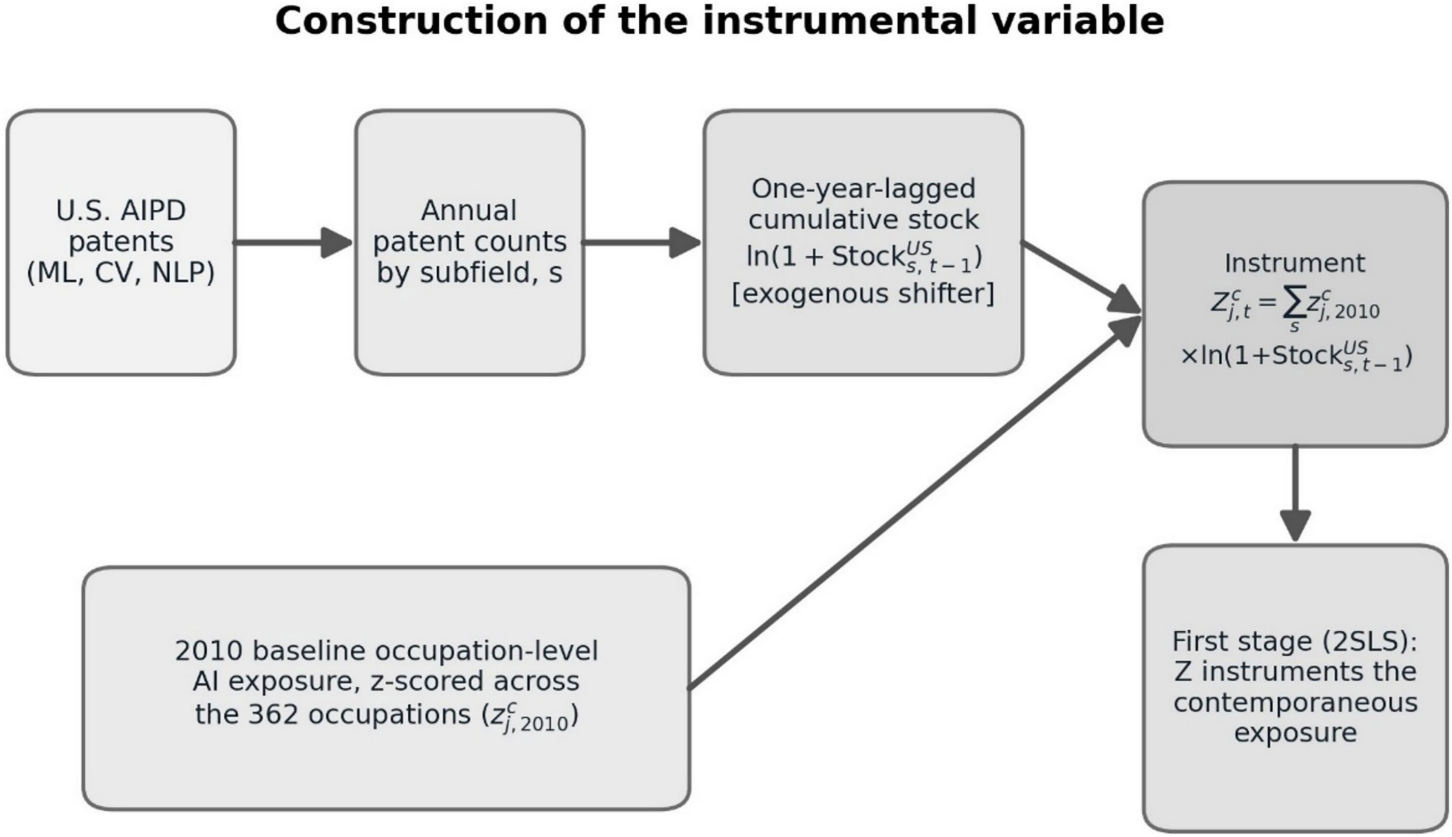
Construction of the instrumental variable. The instrument combines an exogenous time shifter with a predetermined cross-occupation intensity. The shifter is built from U.S. AIPD patent counts in three subfields (machine learning, computer vision, natural language processing), aggregated into a 1-year-lagged cumulative stock. The intensity is the 2010 baseline occupation-level AI exposure (*z*-scored across the 362 five-digit occupations with 2010 exposure data), taken as predetermined from the exposure measure of Section 3.2. Their interaction forms the instrument *Z*, which in the first stage instruments the contemporaneous occupation-level AI exposure.

*Instrumental variable.* The Probit estimates of Equation 2 may be affected by endogeneity from omitted variables, reverse causality, or measurement error in the patent-based exposure proxy. To address these concerns, we construct an instrumental variable that uses variation in AI exposure driven by the external technology frontier. The instrument interacts a predetermined cross-sectional measure of occupation-level AI exposure with a time-varying external shifter. The predetermined component is the cross-sectional z-score of 2010 baseline AI exposure at the five-digit occupation level, computed separately for total, labor-saving (R), and labor-augmenting (N) exposure across all 362 five-digit occupations covered by the 2010 exposure data; the 190 occupations that appear in the CLDS estimation sample (2,446 worker-years) are a subset. Standardization removes scale differences across the three exposure measures, and the 2010 baseline predates the 2012–2018 sample period. The time-varying shifter is the one-year-lagged log cumulative U. S. patent stock in three AI subfields from the USPTO AIPD database: machine learning, natural language processing, and computer vision. For each channel c∈{total,,R,,N} , the aggregate instrument sums across the three subfields:


$$Z_{j,t}^{c}=\underset{s}{\sum }z_{j,2010}^{c}\cdot \ln\left(1+{\mathrm{Stock}}_{s,t-1}^{\mathrm{US}}\right)\;$$ (3)

where the sum runs over the three AI subfields $s\in \left\{\mathrm{ML, NLP, CV}\right\}$ , $z_{j,2010}^{c}$ denotes the standardized 2010 baseline exposure in channel c , and t is defined above. Figure 2 schematically summarizes the construction of the instrument. The baseline IV is just-identified: the total-exposure model instruments total AI exposure with $Z_{j,t}^{total}$ , while the decomposed model instruments labor-saving and labor-augmenting exposure with $Z_{j,t}^{R}$ and $Z_{j,t}^{N}$ , respectively. We estimate the IV specification by two-stage least squares as a linear probability model (LPM), using the same covariates, year and province fixed effects, and origin-occupation-clustered standard errors as in Equation 2. The construction follows the baseline-intensity-times-aggregate-shifter design used in related research (Nunn and Qian, 2014; Acemoglu and Restrepo, 2020; Webb, 2020).

Identification requires that, conditional on the observed covariates and fixed effects, the interaction between lagged U.S. patent stocks and predetermined 2010 occupation-level exposure affects occupational switching only through measured Chinese occupational AI exposure. This restriction cannot be tested directly. Because the U.S. patent-stock shifter varies only over time, year fixed effects absorb its aggregate path and other nationwide shocks common to all occupations, while province fixed effects absorb time-invariant regional differences. Identification therefore comes from occupations’ differential exposure to the common shifter, as determined by their 2010 baseline exposure.

Technology diffusion, multinational investment, and trade are consistent with the exclusion restriction insofar as they affect occupational switching through measured Chinese AI exposure. They threaten the restriction if they generate direct labor-demand or employment effects that bypass measured exposure and covary with occupations’ 2010 exposure intensity. The fixed effects and city-level controls cannot eliminate such time-varying occupation- or industry-specific pathways. We therefore interpret the 2SLS estimates under the maintained exclusion restriction and treat these residual pathways as a limitation.

## 4 Results

### 4.1 Two-channel effects on switching probability

We report Probit average marginal effects as a descriptive baseline in Table 1 and 2SLS linear-probability estimates as our preferred IV specification in Table 2.

**Table 1:** Baseline Probit estimates: average marginal effects.

Variable	(1) Minor switch	(2) Intermediate switch	(3) Major switch
(A) Total AI exposure			
Total AI exposure	0.042***	0.039**	0.007
	(0.015)	(0.016)	(0.019)
Pseudo R^2^	0.071	0.063	0.041
Log-likelihood	−1,427.5	−1,560.2	−1,525.3
(B) Labor-saving and labor-augmenting AI exposure			
Labor-saving AI exposure	0.035**	0.020	−0.015
	(0.016)	(0.015)	(0.023)
Labor-augmenting AI exposure	0.013	0.046**	0.052*
	(0.025)	(0.024)	(0.027)
Pseudo *R*^2^	0.070	0.063	0.044
Log-likelihood	−1,429.5	−1,559.6	−1,520.7
Individual characteristics	Yes	Yes	Yes
City characteristics	Yes	Yes	Yes
Fixed effects	Yes	Yes	Yes
Observations	2,446	2,446	2,446

Average marginal effects (AMEs) from Probit. R = labor-saving (routine-task) exposure; *N* = labor-augmenting (non-routine-task) exposure. All specifications include individual and city controls and year and province fixed effects. Cluster-robust standard errors at the occupation level in parentheses. ***p < 0.01, **p < 0.05, *p < 0.1.

**Table 2:** IV estimates: 2SLS LPM.

Variable	(1) Minor switch	(2) Intermediate switch	(3) Major switch
(A) Total AI exposure			
Total AI exposure	0.036**	0.034**	−0.008
	(0.017)	(0.014)	(0.020)
KP Wald F	72.25	72.25	72.25
Endogeneity test *p*-value	0.559	0.631	0.254
(B) Labor-saving and labor-augmenting AI exposure			
Labor-saving AI exposure	0.022	0.014	−0.040
	(0.021)	(0.017)	(0.028)
Labor-augmenting AI exposure	0.032	0.046*	0.069**
	(0.021)	(0.027)	(0.034)
KP Wald *F*	23.87	23.87	23.87
Endogeneity test *p*-value	0.546	0.784	0.386
Observations	2,446	2,446	2,446

2SLS LPM with the IV constructed in Equation 3. KP Wald *F* = Kleibergen–Paap weak-instrument F-statistic; the endogeneity-test rows report the *p*-value for the null that exposure is exogenous (C-statistic). See Table 1 notes for exposure abbreviations. Cluster-robust standard errors at the occupation level in parentheses. All specifications include individual and city controls and year and province fixed effects. ***p < 0.01, **p < 0.05, *p < 0.1.

*Probit baseline*. Table 1 reports Probit average marginal effects. Total AI exposure is positively associated with minor- and intermediate-group switching but not with major-group switching (Table 1A). In the decomposed estimates (Table 1B), the short-distance association is concentrated in the labor-saving component, while the labor-augmenting component instead strengthens across the wider distance margins.

*IV estimates*. Table 2 reports the 2SLS estimates using the instrument constructed in Equation 3. In Table 2A, a one-log-unit increase in total AI exposure raises minor-group switching by 3.6 percentage points and intermediate-group switching by 3.4 points; the major-group coefficient is statistically indistinguishable from zero. The first stage is strong, with a Kleibergen–Paap Wald F statistic of 72.25. The endogeneity test does not reject exogeneity in any specification (Table 2), and the IV and Probit estimates are similar. We nonetheless report the IV as our preferred specification: it yields a local average treatment effect (LATE) among exposure-responsive occupations and addresses endogeneity and some forms of proxy error under the maintained relevance and exclusion assumptions, which a low-powered endogeneity test need not detect.

In Table 2B, the labor-augmenting coefficients increase from 3.2 percentage points at the minor-group level to 4.6 points at the intermediate-group level and 6.9 points at the major-group level. The latter two coefficients are statistically significant at the 10 and 5% levels, respectively. By contrast, the labor-saving coefficients are statistically indistinguishable from zero at all three distances. The decomposed specification also has adequate first-stage strength, with a Kleibergen–Paap Wald F statistic of 23.87.

Hypothesis H1 is therefore partially supported. The increasing labor-augmenting coefficient pattern is consistent with H1 and with complementary upgrading as a candidate interpretation. H1’s labor-saving prediction appears in the descriptive Probit results but not in the preferred full-sample IV estimates of switching incidence; we therefore treat this evidence as suggestive rather than causal.

### 4.2 Robustness

#### 4.2.1 Lagged exposure and winsorization

To assess sensitivity to exposure timing and extreme values, we re-estimate the IV specifications using exposure dated t−2 and after winsorizing the exposure measures at the 1st and 99th percentiles. Both checks reproduce the baseline pattern: total exposure remains positive at the minor- and intermediate-group levels but not at the major-group level; the labor-augmenting coefficients increase with transition distance, while the labor-saving coefficients remain statistically insignificant. The KP Wald F statistics range from 21.96 to 70.40. Full estimates are reported in Supplementary Table B1.

#### 4.2.2 Propensity-score matching

As a complementary selection-on-observables check, we dichotomize total, labor-saving, and labor-augmenting exposure at their respective sample medians. Propensity scores are estimated using the eight individual and six city-level covariates from the baseline specification together with 6 year indicators. We implement 1:1 nearest-neighbor matching without replacement using a caliper of 0.05 standard deviations of the estimated propensity score (Imbens, 2015) and report inverse-probability-weighted estimates as an additional check. After matching, the maximum absolute standardized mean difference is 0.04.

Table 3 reports the nearest-neighbor ATT estimates. Total exposure has positive ATTs at all three distances, although the positive major-group estimate differs from the null baseline IV result. Because the treatment is defined by a median split, these magnitudes are not directly comparable with the continuous-exposure estimates. In the decomposed results, labor-saving exposure is positive at all three distances, while labor-augmenting exposure is significant only at the intermediate-group level. The PSM evidence therefore supports a positive total-exposure association after balancing observed covariates but does not reproduce the full channel-specific IV pattern. The IPW estimates in Supplementary Table B2 are broadly similar.

**Table 3:** Propensity-score matching: average treatment effects on the treated.

Measure	(1) Minor switch	(2) Intermediate switch	(3) Major switch
ATT — Total AI exposure (high = 1)	0.118***	0.099***	0.089***
	(0.022)	(0.025)	(0.022)
ATT — Labor-saving AI exposure (high = 1)	0.111***	0.099***	0.071***
	(0.023)	(0.024)	(0.024)
ATT — Labor-augmenting AI exposure (high = 1)	0.008	0.075***	0.018
	(0.021)	(0.022)	(0.021)
Matched pairs	904–985	904–985	904–985

Each treatment indicator equals one for workers whose relevant origin-occupation exposure (total, labor-saving, or labor-augmenting) is above its sample median. PSM uses 1:1 nearest-neighbor matching without replacement (caliper = 0.05 SD) on 20 covariates (eight individual, six city-level, 6 year dummies). Bootstrap standard errors (*B* = 200) in parentheses. Post-matching maximum absolute standardized mean difference ≤ 0.04. ***p < 0.01, **p < 0.05, *p < 0.1.

As an exploratory external check, we also examined industry-level robot density, instrumented with rest-of-world robot density following Acemoglu and Restrepo (2020). Because the CLDS occupations map into only five highly uneven International Federation of Robotics (IFR) industry groups, statistical precision is limited. Neither industry-group-clustered inference nor a Webb-weight wild cluster bootstrap designed for few-cluster inference rejects zero at any of the three transition distances. We report the estimates in Supplementary Table B3 but do not use them as evidence for the robustness of the main findings.

### 4.3 Heterogeneity by gender and education

*Gender.* Table 4A reports positive and significant total-exposure coefficients for women at the minor- and intermediate-group margins, whereas the corresponding coefficients for men are statistically insignificant. Men instead exhibit a negative and significant coefficient at the major-group margin. In Table 4B, the positive shorter-distance coefficients for women are concentrated in the labor-augmenting component. For men at the major-group margin, the labor-saving coefficient is negative while the labor-augmenting coefficient is positive, revealing opposing channel-specific effects at this margin.

**Table 4:** Heterogeneity by gender: subsample 2SLS IV estimates.

Variable	(1) Minor switch—F	(1) Minor switch—M	(2) Intermediate—F	(2) Intermediate—M	(3) Major—F	(3) Major—M
(A) Total AI exposure						
Total AI exposure	0.054**	0.021	0.049**	0.022	0.034	−0.046**
	(0.024)	(0.018)	(0.021)	(0.017)	(0.024)	(0.022)
KP Wald *F*	39.86	100.65	39.86	100.65	39.86	100.65
(B) Decomposed AI exposure						
Labor-saving AI exposure	0.023	0.012	0.018	0.005	0.009	−0.087***
	(0.029)	(0.021)	(0.023)	(0.019)	(0.033)	(0.026)
Labor-augmenting AI exposure	0.079*	0.018	0.081**	0.035	0.065	0.078**
	(0.042)	(0.026)	(0.040)	(0.029)	(0.041)	(0.038)
KP Wald *F*	16.24	32.10	16.24	32.10	16.24	32.10
Observations	1,214	1,232	1,214	1,232	1,214	1,232

Subsample 2SLS-IV for women (F) and men (M). Labor-saving and labor-augmenting exposure are instrumented using the instruments constructed in Equation 3. All specifications include individual and city controls and year and province fixed effects. Cluster-robust standard errors at the occupation level in parentheses. Stars denote within-group significance against a null of zero; the table does not report formal tests of coefficient equality across subgroups. ***p < 0.01, **p < 0.05, *p < 0.1.

*Education*. Table 5A shows positive and significant total-exposure coefficients for college-educated workers (years of schooling ≥ 16, *N* = 367) at all three distances. The coefficients are broadly similar across distances and are largest at the major-group margin. Among less-educated workers (*N* = 2,079), only the intermediate-group coefficient is weakly significant, while the major-group coefficient is negative but statistically insignificant. Table 5B shows that labor-saving exposure is positively associated with switching at all three distances among college-educated workers but negatively associated with major-group switching among less-educated workers. For less-educated workers, the labor-augmenting coefficient is positive and significant only at the major-group margin. This combination is consistent with a labor-saving-related barrier to long-distance mobility alongside a possible complementary-upgrading margin, but it does not directly establish either mechanism.

**Table 5:** Heterogeneity by education: subsample 2SLS IV estimates.

Variable	(1) Minor—Low	(1) Minor—High	(2) Intermediate—Low	(2) Intermediate—High	(3) Major—Low	(3) Major—High
(A) Total AI exposure						
Total AI exposure	0.024	0.095***	0.023*	0.094***	−0.024	0.106***
	(0.017)	(0.025)	(0.014)	(0.025)	(0.018)	(0.020)
KP Wald *F*	80.58	110.89	80.58	110.89	80.58	110.89
(B) Decomposed AI exposure						
Labor-saving AI exposure	0.015	0.092***	0.011	0.057*	−0.058**	0.114***
	(0.019)	(0.024)	(0.017)	(0.029)	(0.026)	(0.023)
Labor-augmenting AI exposure	0.022	0.023	0.030	0.082**	0.076**	0.005
	(0.023)	(0.034)	(0.027)	(0.037)	(0.035)	(0.037)
KP Wald *F*	26.79	91.08	26.79	91.08	26.79	91.08
Observations	2,079	367	2,079	367	2,079	367

Subsample 2SLS-IV estimates. Low education = years of schooling <16; High education = years of schooling ≥ 16 (bachelor’s degree or above). Labor-saving and labor-augmenting exposure are instrumented using the instruments constructed in Equation 3. All specifications include individual and city controls and year and province fixed effects. Cluster-robust standard errors at the occupation level in parentheses. Stars denote within-group significance against a null of zero; the table does not report formal tests of coefficient equality across subgroups. ***p < 0.01, **p < 0.05, *p < 0.1.

Taken together, the positive total-exposure switching responses are concentrated among women and college-educated workers, whereas less-educated workers show a negative labor-saving coefficient at the major-group margin. Because the tables do not report formal tests of cross-group coefficient equality, and because the college-educated sample is relatively small, these differences should be interpreted as exploratory. Section 5.2 discusses their potential implications for targeted labor-market policy.

### 4.4 Where switchers go and how they fare

This section shifts from switching incidence to analyses conditional on switching: among workers who switch, where do they move (Section 4.4.1) and how do they fare (Section 4.4.2)? The total-exposure measure can mask channel-specific patterns, so the discussion below focuses on the decomposed IV estimates. Because switching is itself affected by AI exposure, conditioning on observed switchers introduces endogenous selection. We therefore interpret the estimates in this section as selected-sample relationships among switchers rather than as the same LATE identified for switching incidence in Section 4.1.

#### 4.4.1 Origin–destination exposure sorting

**Figure 3:**
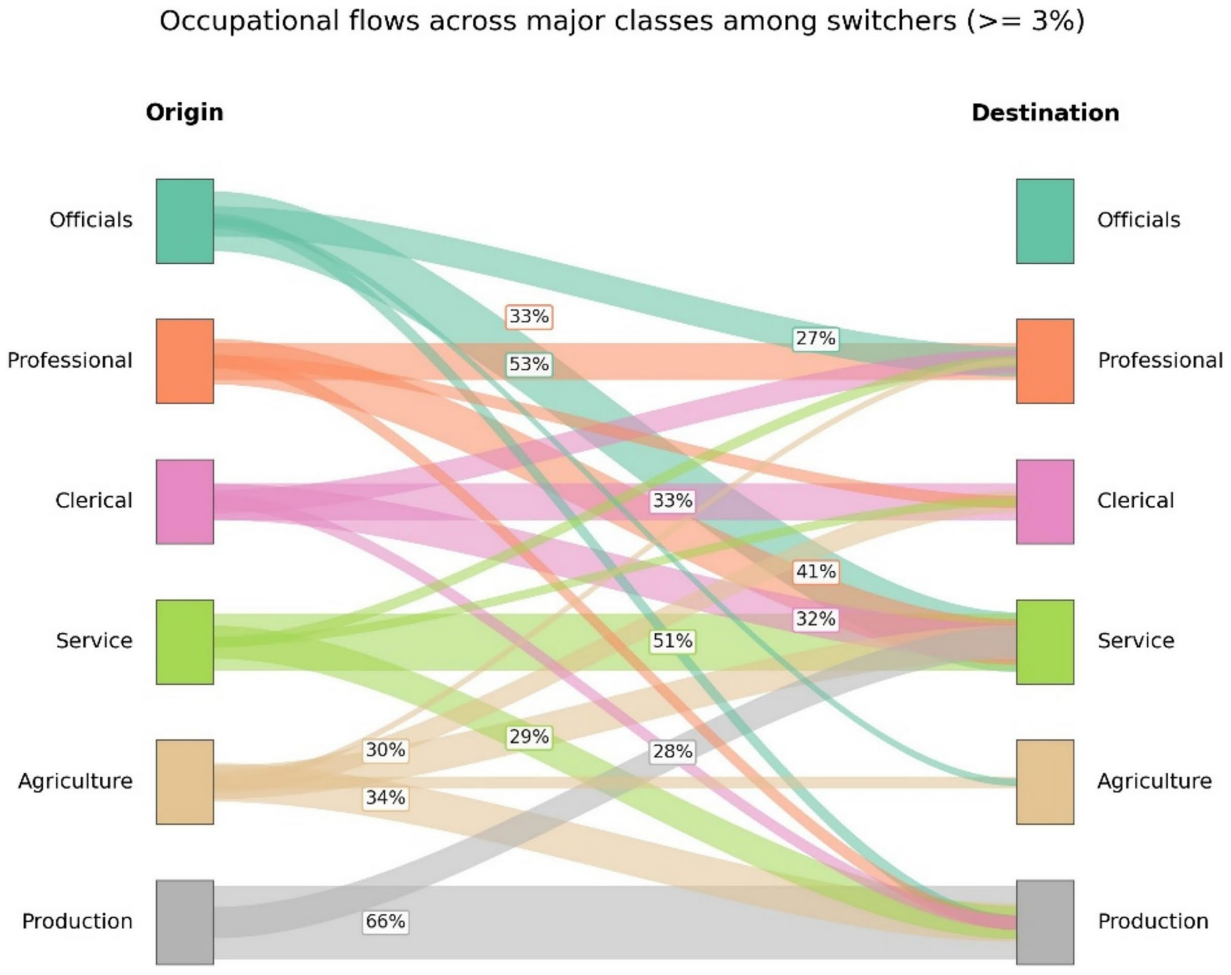
Sankey diagram of major-group occupational flows. Occupational flows across major groups for workers who switched at the minor-group level (*N* = 1,659). Ribbon width is proportional to the transition share; labelled percentages give each origin group’s largest flows (as a share of the origin group). All flows ≥3% of the origin group are drawn.

Figure 3 plots how the minor-group switchers are distributed across major occupational groups. Most remain within their origin major group (for example, 66% of production-origin and 51% of service-origin switchers), so switching is predominantly short-distance, which motivates the nested transition-distance design used below. Among those who do cross major groups, destinations are uneven: service is the largest net-inflow group, and production-to-service is the dominant cross-group flow. The diagram is descriptive; Table 6 then characterizes origin–destination exposure sorting among switchers, and Table 7 reports their post-transition job conditions.

**Table 6:** Origin–destination exposure sorting among switchers: decomposed 2SLS estimates.

Variable	(1) Minor: destination R	(2) Minor: destination N	(3) Intermediate: destination R	(4) Intermediate: destination N	(5) Major: destination R	(6) Major: destination N
Origin labor-saving AI exposure	0.128*** (0.042)	−0.022 (0.042)	0.059 (0.044)	−0.007 (0.043)	−0.238*** (0.055)	−0.134*** (0.041)
Origin labor-augmenting AI exposure	0.083 (0.065)	0.238*** (0.069)	0.077 (0.071)	0.152** (0.069)	0.202** (0.094)	0.196*** (0.068)
*R*^2^	0.216	0.132	0.187	0.110	0.126	0.075
KP Wald *F*	26.67	26.67	25.24	25.24	28.31	28.31
Observations	1,659	1,659	1,416	1,416	867	867

2SLS estimates for three nested samples of workers who switched at the minor-, intermediate-, and major-group margins. Outcomes are destination-occupation log labor-saving (R) and labor-augmenting (N) exposure. Origin R and N exposure are jointly instrumented using the instruments constructed in Equation 3. Stars test the null that a coefficient equals zero (no origin–destination sorting); a coefficient of one would indicate one-for-one persistence, and all six own-dimension slopes reject a slope of one at *p* < 0.01. All specifications include individual and city controls and year and province fixed effects. Cluster-robust standard errors at the origin-occupation level are in parentheses. Total-exposure estimates are reported in Supplementary Table B4; descriptive within-worker direction indicators are reported in Supplementary Table B5. ***p < 0.01, **p < 0.05, *p < 0.1.

**Table 7:** Post-transition job conditions among switchers: decomposed 2SLS estimates.

Variable	Weekly hours			Skill-match satisfaction		
	Minor	Inter.	Major	Minor	Inter.	Major
Origin labor-saving AI exposure	0.960* (0.493)	0.853* (0.505)	0.847 (0.683)	−0.033** (0.016)	−0.036* (0.019)	−0.044* (0.024)
Origin labor-augmenting AI exposure	−1.249** (0.622)	−0.821 (0.626)	−0.915 (0.815)	0.047* (0.025)	0.043 (0.027)	0.052* (0.031)
KP Wald *F*	26.67	25.21	28.31	26.75	25.52	28.77
Observations	1,657	1,415	867	1,590	1,355	834

Selected-sample 2SLS estimates for three nested samples of workers who switched at the minor-, intermediate-, and major-group margins. Origin labor-saving and labor-augmenting exposure are jointly instrumented using the instruments constructed in Equation 3. Weekly hours is continuous; skill-match satisfaction is a binary outcome estimated as a linear-probability model. Sample sizes vary across outcomes because of item non-response. All specifications include individual and city controls and year and province fixed effects. Cluster-robust standard errors at the origin-occupation level are in parentheses. Total-exposure counterparts are reported in Supplementary Table B7; destination-job training-requirement estimates are reported in Supplementary Table B8. ***p < 0.01, **p < 0.05, *p < 0.1.

We estimate destination-sorting regressions in three nested switching samples: minor-group (*N* = 1,659), intermediate-group (*N* = 1,416), and major-group (*N* = 867). The outcomes are destination-occupation labor-saving and labor-augmenting exposure, and the regressors of interest are the corresponding origin exposures, jointly instrumented as in Equation 3. The slope summarizes how much of the exposure gap between workers at their origin occupations is preserved at their destinations: a slope of one means origin differences carry over fully, a slope between zero and one means workers from more exposed origins still reach relatively more exposed destinations but the gap between them narrows, and a slope of zero means destination exposure is unrelated to origin exposure. Because the samples are nested, the coefficients describe sorting within each switching margin rather than within mutually exclusive distance bins. The total-exposure counterparts appear in Supplementary Table B4, and descriptive within-worker exposure-change indicators in Supplementary Table B5.

Table 6 shows that all six own-dimension slopes reject the unit-slope benchmark, ruling out one-for-one transmission of origin-exposure differences to destination occupations. On the labor-saving dimension, sorting is significantly positive in the minor-group sample, statistically indistinguishable from zero in the intermediate-group sample, and significantly negative in the major-group sample. On the labor-augmenting dimension, sorting is positive and significant in all three samples, indicating positive but attenuated persistence. The cross-dimension slopes are statistically insignificant in the minor- and intermediate-group samples. At the major-group margin, however, higher origin labor-saving exposure is associated with lower destination labor-augmenting exposure, while higher origin labor-augmenting exposure is associated with higher destination labor-saving exposure.

These estimates give H2 partial support. The negative labor-saving sorting predicted by a defensive-sorting account appears only in the major-group sample; the minor-group coefficient is positive and the intermediate-group coefficient is not statistically distinguishable from zero. Positive labor-augmenting sorting in all three samples is consistent with a portable-skill mechanism. The positive coefficient remains significant in the major-group sample, where within-major-group occupational similarity cannot account for the result, strengthening but not establishing the portable-skill interpretation. Nor does the evidence rule out complementary upgrading, which does not require positive sorting. Throughout, these slopes describe sorting between workers: a negative slope means that workers from more exposed origins enter relatively less-exposed destinations than workers from less-exposed origins, not that a given worker’s exposure necessarily falls. Establishing active avoidance would require a benchmark of feasible destinations or direct behavioral evidence that the data do not provide.

Supplementary Table B6 provides a descriptive counterpart. Across initial total-exposure tertiles in the minor-group switching sample, mean destination exposure rises monotonically on both dimensions, but the differences are substantially smaller than the corresponding origin-exposure differences. This pattern is consistent with attenuated origin–destination sorting but is not a causal validation of the IV estimates. In career-adaptation terms (Savickas, 2013; Spurk et al., 2019), negative labor-saving sorting in the major-group sample is consistent with a defensive-sorting interpretation at that margin. Positive labor-augmenting sorting is consistent with skill portability but does not by itself establish exploratory adaptation.

#### 4.4.2 Post-transition job conditions

We focus on two post-transition conditions that map directly onto the organizational-psychology framework: weekly working hours, which capture one quantitative dimension of job demands, and self-reported skill-match satisfaction, which we interpret as an indicator of perceived person–job fit. We estimate the decomposed 2SLS specification in the same three switching samples as in Section 4.4.1. The regressors of interest are origin-occupation labor-saving and labor-augmenting exposure, jointly instrumented as in Equation 3. Table 7 reports the decomposed estimates, while total-exposure counterparts appear in Supplementary Table B7. Sample sizes vary across outcomes because of item non-response. Estimates for whether the destination job requires training are reported separately in Supplementary Table B8 because the welfare interpretation of that outcome is ambiguous: a training requirement may indicate either a skill gap or entry into a more skill-intensive job.

Origin labor-saving exposure is positively associated with weekly hours in the minor- and intermediate-group samples, although both estimates are significant only at the 10% level; the major-group estimate is statistically insignificant. Skill-match satisfaction is negatively associated with labor-saving exposure in all three samples, with significance at the 5% level in the minor-group sample and at the 10% level in the intermediate- and major-group samples.

Origin labor-augmenting exposure is associated with shorter weekly hours in the minor-group sample, but the intermediate- and major-group estimates are statistically insignificant. Its skill-match coefficient is positive in all three samples and significant at the 10% level in the minor- and major-group samples, but statistically insignificant in the intermediate-group sample. The total-exposure coefficients in Supplementary Table B7 are statistically insignificant for both outcomes, consistent with aggregation masking some channel-specific associations.

Taken together, these selected-sample estimates provide partial support for Hypothesis H3. The most consistent evidence concerns the negative association between labor-saving exposure and perceived skill match. Evidence for longer hours and for favorable labor-augmenting outcomes is confined to specific switching samples. These outcomes capture particular aspects of destination-job fit and demands rather than overall job quality or worker well-being.

## 5 Discussion

### 5.1 Distance-channel patterns and possible mechanisms

The distance-channel patterns indicate that AI exposure is not associated with a uniform mobility response. In the full-sample switching-incidence estimates, evidence for the labor-saving channel remains suggestive because its IV coefficients are not statistically distinguishable from zero. The subgroup estimates show negative labor-saving coefficients for men’s and less-educated workers’ major-group switching, whereas the conditional-on-switching analyses show margin-specific destination sorting and post-transition associations among observed switchers. In combination, these results point to stratified labor-market adjustment rather than establishing a general displacement effect of labor-saving exposure on occupational switching.

One possible interpretation of the labor-augmenting distance gradient is complementary upgrading: AI exposure may broaden the set of distant occupations accessible to some workers. The evidence does not identify this pathway directly. Positive selection into distant moves or pre-existing human capital could generate the same pattern, and the data contain no worker-level measures of AI adoption, cognitive-skill acquisition, or learning. Positive origin–destination sorting on the labor-augmenting dimension is also consistent with portable skills, but it does not establish exploratory adaptation or rule out complementary upgrading. We therefore retain complementary upgrading and skill portability as candidate interpretations rather than identified mechanisms.

Cross-country evidence likewise suggests that workers’ responses to technological change vary with their initial skill stocks and adjustment capacity (Cazzaniga et al., 2024; Cortes, 2016). In China, the estimates are more consistent with heterogeneous than uniform adjustment across education groups and switching distances, although the study does not directly measure adjustment capacity and does not formally test cross-group coefficient differences.

In career-adaptation terms, the two channels carry different behavioral logics, though the data do not identify workers’ motives directly. Viewed through the defensive lens, the margin- and subgroup-specific labor-saving estimates summarized above are consistent with displacement pressure constraining long-distance mobility for specific groups. The negative major-group destination-sorting estimate is likewise consistent with movement toward destinations with relatively lower labor-saving exposure, although it does not reveal the motive behind such moves. Related recent U.S. evidence cautions that mobility need not imply successful adjustment: workers leaving declining, highly AI-exposed occupations disproportionately enter other declining occupations (Song et al., 2026).

Viewed through the exploratory lens, the rising labor-augmenting gradient and the positive destination sorting are consistent with an expanding opportunity set. The two candidate pathways discussed above also help interpret the distance gradient. Portable skills or complementary upgrading may ease the skill constraints on longer transitions, whereas labor-saving exposure does not itself relax those constraints. Neither reading separates motives from constraints; we therefore treat defensive and exploratory adaptation as interpretive lenses rather than tested mechanisms.

For organizational psychology, the contribution lies in examining how instrumented occupation-level AI exposure relates to observable career-adaptation behavior and destination-job conditions, adding behavior-level evidence to resource-focused accounts of career adaptability.

### 5.2 Implications for organizations and policy

The negative labor-saving coefficient for major-group switching among less-educated workers suggests that less-educated workers in occupations with high labor-saving exposure may warrant particular attention from organizations and policymakers. This implication remains tentative because the subgroup differences are exploratory and our exposure measure captures potential AI applicability rather than realized adoption. Evidence from a specific customer-support setting shows that generative-AI assistance can produce larger productivity gains for novice and lower-skill workers (Brynjolfsson et al., 2025). Although that setting and technology differ from those examined here, the result supports testing training and tool designs that help less-experienced workers use AI complementarily; it does not establish that upskilling reverses labor-saving displacement. More broadly, Bessen (2019) emphasizes that the employment consequences of productivity-enhancing technologies depend on the demand response, while Acemoglu et al. (2020) show that tax systems can create excessive incentives for automation relative to labor.

AI adoption also changes the skills employers demand: AI-adopting establishments alter the skill requirements of their job postings (Acemoglu et al., 2022). These changing requirements create employer-side incentives to supply training, because skill gaps can constrain adoption itself: in seven-country OECD surveys, about two in five employers that had not adopted AI cited a lack of relevant skills as a barrier (Lane et al., 2023). Firm-provided training is also theoretically plausible because imperfect labor markets allow employers to capture part of the returns, even when the skills are general (Acemoglu and Pischke, 1999). Consistent with this mechanism, a majority of surveyed workers using AI reported that their employer had provided or funded AI-related training (Lane et al., 2023).

In China, the institutional basis for offering AI-complementary training before displacement pressures materialize already exists. The 2022 revision of the Vocational Education Law provides a general legal basis for vocational training and apprenticeship, covering newly hired workers, incumbent workers, and workers moving between posts. The 14th Five-Year Plan for Employment Promotion likewise calls for worker skills to match industrial upgrading more closely, and it supports training for incumbent workers, for workers changing occupations, and for building skills ahead of demand.

For organizations, the findings suggest four potential levers. Internal-mobility and career-counseling systems can help workers identify skills-adjacent or AI-complementary roles. On-the-job AI training can be made accessible to workers with fewer formal qualifications. Job redesign can pair AI tools with complementary human tasks. Finally, working hours and perceived skill match, the two destination-job conditions examined in Section 4.4.2, are worth monitoring during technology-related transitions. These recommendations, including the emphasis on pre-displacement timing, follow from exploratory subgroup estimates and selected-sample associations, not from direct intervention evidence. Evaluating and prioritizing such interventions would require a formal welfare analysis that accounts for the kind of geographically concentrated employment and wage effects documented in highly exposed local labor markets (Acemoglu and Restrepo, 2020), which remains for future research.

### 5.3 Limitations

Six limitations bear on the interpretation of our results.

First, both the exposure measure and the switching outcome are subject to measurement concerns. The patent-based exposure measure captures potential task-level applicability rather than realized workplace adoption. This conceptual gap may generate measurement error or proxy mismatch whose direction cannot be signed, so the estimates should not be read as conservative lower bounds; the IV strategy mitigates only some forms of proxy error, and only under the maintained relevance and exclusion assumptions. On the outcome side, changes in five-digit occupational codes may reflect moves between closely related occupations or reporting and coding differences rather than economically substantial transitions. Because such moves and most reporting or coding differences are unlikely to cross major-group boundaries, results at the broader switching margins provide a partial check, but they do not eliminate this concern.

Second, the subgroup results should be interpreted as exploratory. The college-educated subsample includes only 367 workers, and coefficient equality across education or gender groups is not formally tested. Consequently, differences in coefficient signs or statistical significance across subgroups should not be treated as established cross-group differences.

Third, causal interpretation of the 2SLS estimates rests on an untestable exclusion restriction. The residual threat, discussed in Section 3.3, is labor-demand effects that bypass measured AI exposure and covary with occupations’ baseline exposure, as could arise from technology diffusion, multinational investment, or trade shocks; fixed effects and city-level controls cannot rule these out. Moreover, while the switching-incidence estimates in Section 4.1 carry a LATE interpretation under these assumptions, the destination-sorting and post-transition analyses in Section 4.4 additionally condition on observed switchers and therefore describe selected-sample relationships.

Fourth, the CLDS does not contain validated multi-item measures of career adaptability, person–job fit, Job Demands–Resources constructs, or psychological well-being. Occupational switching is therefore interpreted as observable career-adaptation behavior rather than as a measure of adaptability resources; skill-match satisfaction is a limited indicator of perceived fit, and weekly hours capture only one quantitative job demand. These proxies do not support conclusions about multidimensional job quality or overall worker well-being. Future research should combine technology-exposure measures with validated psychological scales and longitudinal well-being outcomes.

Fifth, the sample period ends in 2018 and therefore predates the diffusion of current generative large language models. Generative models extend potential AI applicability into open-ended cognitive and creative tasks (Eloundou et al., 2024). Whether this strengthens the labor-augmenting channel is not yet clear: field studies document productivity gains in some organizational settings (Brynjolfsson et al., 2025), while early economy-wide evidence shows little change in earnings or working hours (Humlum and Vestergaard, 2025). At the career-entry margin, however, search intensity for Swiss apprenticeship vacancies declined in the short and medium term following ChatGPT’s launch, particularly for occupations intensive in cognitive tasks and language skills (Goller et al., 2025). The external validity of these estimates for the generative-AI era therefore remains an open question.

Sixth, the evidence comes from a single country, so the estimates should not be read as universal adjustment parameters. Cross-country studies show that AI exposure and the balance between automation and augmentation vary with national occupational structures and income levels (Gmyrek et al., 2023; Cazzaniga et al., 2024). Beyond differences in exposure, institutions may shape the conversion of exposure into mobility: training systems, internal labor markets, employment protection, and mobility-support policies can affect workers’ ability to acquire AI-complementary skills or move across occupations. Job mobility itself responds to structural change in systematically different ways across institutional regimes (DiPrete et al., 1997). Cross-national research on career adaptability likewise shows that career-adaptation resources can be measured comparably across countries, while leaving room for institutional and cultural variation in how they are expressed (Savickas and Porfeli, 2012). Cultural norms about occupational mobility and employer responsibility for training may further influence whether adjustment occurs through internal redeployment, external switching, or delayed mobility. Early comparative evidence points to both commonality and heterogeneity. In Brazil and the United Kingdom, college-educated workers show broadly similar historical transitions from high-exposure, low-complementarity occupations toward high-exposure, high-complementarity ones. Non-college workers in Brazil, however, face greater risks of downward mobility (Cazzaniga et al., 2025). Further comparative evidence is therefore needed to assess whether the Chinese patterns estimated here extend to other institutional and cultural settings.

## 6 Conclusion

This study separates the labor-saving and labor-augmenting channels of occupational AI exposure and documents distinct mobility patterns in China. Under the maintained IV assumptions, a one-log-unit increase in total AI exposure raises minor-group switching by 3.6 percentage points and intermediate-group switching by 3.4 points, while the major-group coefficient is statistically indistinguishable from zero. The decomposed labor-augmenting coefficients rise with transition distance and are significant at the intermediate- and major-group margins, reaching 6.9 percentage points at the major-group margin, where the labor-saving coefficient is negative but imprecisely estimated. In the selected samples of observed switchers, labor-augmenting exposure exhibits positive but attenuated origin–destination sorting at all three margins. Labor-saving sorting is positive in the minor-group sample, statistically indistinguishable from zero in the intermediate-group sample, and negative in the major-group sample. The post-transition estimates also vary across the switching samples: origin labor-saving exposure is associated with longer hours in the minor- and intermediate-group samples and with lower skill-match satisfaction in all three samples, whereas favorable origin labor-augmenting associations are confined to specific samples. These sorting and post-transition estimates are selected-sample relationships rather than the switching-incidence LATE.

The subgroup estimates suggest a labor-saving-related barrier to major-group mobility among less-educated workers. Because these differences are exploratory and not supported by formal cross-group coefficient tests, we read this result as flagging less-educated workers in occupations with high labor-saving exposure as a group that may warrant particular attention, not as establishing a policy target. For organizational psychology, the broader implication is that adaptation to technological change should be evaluated both by whether workers move and by the destination-job conditions associated with those moves.

Relative to the three stylized facts reviewed in Section 1, the results offer three refinements. First, the total-exposure coefficient remains statistically indistinguishable from zero at the major-group margin, while the decomposition reveals a significant positive labor-augmenting coefficient and an imprecisely estimated labor-saving coefficient; an aggregate null therefore need not indicate the absence of channel-specific responses. Second, earlier evidence of adverse adjustment among routine-task workers (Cortes, 2016; Feigenbaum and Gross, 2024) is echoed here only for specific groups and margins: labor-saving exposure predicts lower major-group mobility among less-educated workers and is associated with less favorable destination-job conditions among switchers in specific samples. These estimates do not establish a common downgrading trajectory or show that the patterns concern the same workers. Third, the distinction between automation-oriented and augmentation-oriented AI (Autor et al., 2024) corresponds closely to the labor-saving and labor-augmenting channels separated here. The labor-augmenting distance pattern is consistent with complementary upgrading, while positive labor-augmenting origin–destination sorting is consistent with portable skills; the design does not distinguish between these candidate interpretations.

Accordingly, all three hypotheses receive partial support. For H1, the labor-augmenting coefficients exhibit a positive distance pattern consistent with the complementary-upgrading prediction, whereas the labor-saving coefficients are statistically indistinguishable from zero in the full-sample switching-incidence IV estimates. For H2, negative labor-saving sorting appears only in the major-group sample, while positive labor-augmenting sorting appears in all three samples and is consistent with, but does not establish, skill portability. For H3, origin labor-saving exposure is associated with lower skill-match satisfaction in all three switching samples and with longer hours in the minor- and intermediate-group samples; favorable origin labor-augmenting associations are confined to specific samples.

Five extensions are priorities for future research. First, replication with post-2022 data is needed because generative AI extends potential exposure into new cognitive tasks and its channel-specific mobility effects may differ materially from those observed during 2012–2018. Second, linking patent-based exposure to firm-level adoption data would tighten the connection between potential applicability and realized AI use. Third, worker-level skill and learning measures would help distinguish complementary upgrading from portable-skill sorting. Fourth, longer pre-switching worker histories, together with credible predictors of selection into switching, would support explicit selection models and sensitivity analyses for the conditional-on-switching estimates. Fifth, linking micro-level switching to regional and national labor-market outcomes would permit an assessment of the aggregate socio-economic costs and benefits of AI-related occupational reallocation. Relevant dimensions include local employment, wage inequality, training costs, productivity gains, and the distribution of adjustment burdens across regions and, with comparable data, across countries.

## Data Availability

The data analyzed in this study is subject to the following licenses/restrictions: the individual-level CLDS microdata are not publicly available. Access is restricted and requires an application to the data provider, the Center for Social Survey at Sun Yat-sen University, under its data use policy. The Chinese AI patent texts were retrieved from Patyee, a commercial patent search-and-analytics database that requires institutional subscription. The industrial robot data analyzed in the Supplementary material are from the International Federation of Robotics (IFR) World Robotics database, which requires commercial purchase. Requests to access these datasets should be directed to CLDS: Center for Social Survey, Sun Yat-sen University, https://css.sysu.edu.cn; Patyee: https://www.patyee.com; IFR World Robotics: https://ifr.org.
