# Supplementary material for: AI exposure, occupational mobility, and post-transition job quality in China: labor-saving and labor-augmenting channels
Source: Front Psychol. 2026 Jul 28;17:1799093. doi: 10.3389/fpsyg.2026.1799093 (PMC13457157; doi:10.3389/fpsyg.2026.1799093)
Supplement: Supplementary file 1 [file Data_Sheet_1.docx]

# 1 Supplementary Material: supplementary methods, validation and robustness analyses, Supplementary Figures A1–A2, Supplementary Table A1, Supplementary Tables B1–B8, and the full AI-patent keyword list.

## 1.1 SI Section A — Exposure Construction Algorithm and Descriptive Statistics

### 1.1.1 A.1 Full exposure construction algorithm

Building on the brief summary in main-text Section 3.2, the full three-step construction is as follows.

**Step 1: Text vectorization.** Both task statements and patent texts are encoded into 384-dimensional dense vectors using the paraphrase-multilingual-MiniLM-L12-v2 model from the sentence-transformers framework. All vectors are $L_{2}$-normalized so that the inner product of any two vectors equals their cosine similarity.

**Step 2: Similarity computation and thresholding.** For each year $t$, FAISS (Johnson et al., 2021) is used to build an inner-product index over the year’s patent vectors. For each task statement $i$, the top $K=50$ most similar patents are retrieved. Because the threshold defined below sets all sub-threshold patents to zero, patents beyond rank 50 typically fall below the threshold and add negligible mass. Consistent with this, raising *K* from 50 to 70 changes occupation-level exposure by less than 0.3% (Pearson correlation at least 0.997 across occupation-years). Scores are then sparsified at a year-specific threshold $\tau_{t}$ defined as the 60th percentile of the top-$K$ maxima across all task statements. This follows the percentile-sparsification design of Kogan et al. (2023), recalibrated to the Chinese-language corpus and multilingual embeddings, and is lower than their 80th-percentile cutoff on the full patent-occupation similarity distribution. Robustness to both choices is examined in Section A.4:

$$\tau_{t}=Q_{0.60}(\{\max_{k}s_{i,k}^{(t)}:i\in\mathcal{I}_{n}\}) (\text{S1})$$

The truncated similarity is

$$\tilde{s}_{i,k}=max(0,s_{i,k}-\tau_{t}) (\text{S2})$$

and the task-level AI relatedness score is

$$x_{i,t}=\sum_{k=1}^{K} \tilde{s}_{i,k} (\text{S3})$$

**Step 3: Occupation-level aggregation.** As reported in main-text Equation (1), occupation-level exposure measures are constructed by summing task-level scores over routine, non-routine, and the union of task statements within each occupation.

### 1.1.2 A.2 Validation of the task classification

**Manual validation.** To assess the correspondence between the GPT-3.5 labels and human judgment, two independent human coders classified the same blind random sample of 250 task statements drawn from the full set of 9,334 statements, using the same task-content definition and without access to the model labels. The routine shares assigned by the two coders were 58.0% and 58.8%, compared with 63.6% for GPT-3.5 in the same sample, 62.6% for GPT-3.5 in the full corpus, and the 62% reported by Kogan et al. (2023) for U.S. occupations under the DOT classification. Inter-rater agreement between the two human coders was 86.4% (Cohen’s κ = 0.720). Across the two coders, agreement with GPT-3.5 ranged from 79.2% to 80.0% (κ = 0.565–0.580), while agreement with GLM-4.6 was 80.4% for both coders (κ = 0.579–0.582). The close routine shares and comparable human–model agreement across coders indicate that the validation results are not driven by a single human judgment.

**Cross-model agreement.** To assess whether the classification depends on the specific large language model used, we re-label all 9,334 task statements with Zhipu GLM-4.6 under an identical prompt template. Agreement between GPT-3.5 and GLM-4.6 is 80.0% (Cohen’s κ = 0.558). Replacing the GPT-3.5 labels with the GLM-4.6 labels leaves occupation-level exposure and the resulting IV estimates substantively unchanged (Section A.4).

### 1.1.3 A.3 Descriptive Statistics

Supplementary Table A1 reports summary statistics for the core variables. The minor-, intermediate-, and major-group switching means are 0.678, 0.579, and 0.354 in the baseline IV sample (N = 2,446). Mean log total AI exposure is 1.694, with the labor-saving component (1.539) substantially larger and more spread than the labor-augmenting component (0.544). Average age is 39.0 years, 50.4% male, 11.3 years of schooling, 82.7% married, and 47.5% non-agricultural *hukou*. Among occupation major groups, production and manufacturing has the highest total exposure (2.90), driven almost entirely by the labor-saving component, while professional and technical occupations are the only major group in which labor-augmenting exposure exceeds labor-saving. Across the 190 unit groups in the sample, labor-saving and labor-augmenting exposure are correlated (ρ = 0.48) but retain independent variation in their R–N relative structure, motivating the decomposition. Supplementary Figure A1 plots the kernel density of the three exposure measures conditional on positive exposure, showing that the labor-saving distribution closely tracks the total distribution while the labor-augmenting distribution is concentrated at lower values and has a substantially larger zero mass (22.6% zeros versus 11.9% for labor-saving).

**Supplementary Figure A1**. *Kernel density of AI exposure measures, 2010–2018.* Kernel density of log total, labor-saving R, and labor-augmenting N AI exposure conditional on positive exposure. Source: authors’ calculations.

**Supplementary Table A1.** *Summary statistics for core variables (N = 2,446).*

| Variable | Mean | Std. Dev. | Min | Max |
| --- | --- | --- | --- | --- |
| **Dependent variables** |  |  |  |  |
| Minor-group switching (=1) | 0.678 | 0.467 | 0.000 | 1.000 |
| Intermediate-group switching (=1) | 0.579 | 0.494 | 0.000 | 1.000 |
| Major-group switching (=1) | 0.354 | 0.478 | 0.000 | 1.000 |
| **AI exposure (log)** |  |  |  |  |
| Total | 1.694 | 1.219 | 0.000 | 4.918 |
| Labor-saving | 1.539 | 1.221 | 0.000 | 4.893 |
| Labor-augmenting | 0.544 | 0.670 | 0.000 | 3.588 |
| **Individual characteristics** |  |  |  |  |
| Age | 38.956 | 10.949 | 16.000 | 65.000 |
| Male (=1) | 0.504 | 0.500 | 0.000 | 1.000 |
| Years of education | 11.256 | 3.703 | 0.000 | 19.000 |

*Notes*: AI exposure variables are in log units. Additional individual covariates (marital status, health status, Communist Party membership, *hukou* status) and the six city-level covariates included in all specifications are dropped from this summary table but enter all specifications as controls. Sample-specific switcher counts: 1,659 minor-group switchers, 1,416 intermediate-group switchers, and 867 major-group switchers; the nested structure is confirmed in the data.

### 1.1.4 A.4 Robustness to exposure-construction choices

To examine whether the results depend on exposure-construction parameters, we re-estimate the main IV specification over a one-at-a-time grid of retrieval caps and sparsification thresholds: K ∈ {30, 50, 70} holding $\tau_{q}$= 0.60, and $\tau_{q}$ ∈ {0.50, 0.60, 0.70, 0.80} holding K = 50, where 0.80 corresponds to the cutoff used by Kogan et al. (2023). We also report the GLM-4.6-labelled variant described in Section A.2. The baseline configuration reproduces the main-text Table 2 estimates exactly. Across these alternative constructions, no coefficient changes sign, and every alternative point estimate lies within the baseline 95% confidence interval (Supplementary Figure A2), so variation due to construction choices is smaller than baseline sampling uncertainty. The increasing labor-augmenting gradient over transition distances is preserved in all six parameter configurations; under the GLM-4.6-labelled variant, the major-margin labor-augmenting coefficient remains the largest and most significant, while several individual coefficients cross conventional significance thresholds.

The analysis therefore shows that the conclusions do not depend on the particular values of K or $\tau_{q}$, but it does not imply that the baseline values are uniquely correct. Those values are motivated by the precedent and corpus-specific recalibration in Section A.1, and the reliability of the routine/non-routine labels through the inter-rater, human–model, and cross-model agreement evidence in Section A.2.

**Supplementary Figure A2**. *Specification curve: IV estimates across exposure-construction choices.* Each panel plots the IV point estimate and 95%  confidence interval for one dependent variable (minor/intermediate/major switching) × one exposure term (total, labor-saving R, labor-augmenting N) across the grid (K ∈ {30, 50, 70}; τ ∈ {0.50, 0.60, 0.70, 0.80}) and the GLM-4.6-labelled variant. The shaded band is the baseline (K = 50, τ = 0.60) 95% confidence interval; every alternative estimate lies within it. The red dashed line marks zero.

## 1.2 SI Section B — Auxiliary Tables

### 1.2.1 Supplementary Table B1 — Sensitivity analyses: lagged exposure and winsorization

| Variable | (1) Minor switch | (2) Intermediate switch | (3) Major switch |
| --- | --- | --- | --- |
| **Panel A: Lagged exposure — total** | | | |
| Total AI exposure (t-2) | 0.034** | 0.033** | −0.008 |
|  | (0.016) | (0.014) | (0.019) |
| KP Wald F | 58.73 | 58.73 | 58.73 |
| **Panel B: Lagged exposure — decomposed** | | | |
| Labor-saving AI exposure (t-2) | 0.021 | 0.013 | −0.039 |
|  | (0.020) | (0.016) | (0.028) |
| Labor-augmenting AI exposure (t-2) | 0.029 | 0.043* | 0.066** |
|  | (0.020) | (0.025) | (0.032) |
| KP Wald F | 21.96 | 21.96 | 21.96 |
| **Panel C: Winsorized — total** | | | |
| Total AI exposure (winsorized) | 0.036** | 0.035** | −0.008 |
|  | (0.018) | (0.014) | (0.020) |
| KP Wald F | 70.40 | 70.40 | 70.40 |
| **Panel D: Winsorized — decomposed** | | | |
| Labor-saving AI exposure (winsorized) | 0.022 | 0.014 | −0.041 |
|  | (0.021) | (0.017) | (0.029) |
| Labor-augmenting AI exposure (winsorized) | 0.033 | 0.048* | 0.073** |
|  | (0.022) | (0.028) | (0.035) |
| KP Wald F | 22.70 | 22.70 | 22.70 |
| Observations | 2,446 | 2,446 | 2,446 |

*Notes*: 2SLS LPM. Panels A–B use AI exposure lagged by two periods; Panels C–D use AI exposure winsorized at the 1st and 99th percentiles. Cross-referenced from main-text Section 4.2.1. Exposure is instrumented using the instruments constructed in Eq. (3); all specifications control for individual and city characteristics, include year and province fixed effects, and cluster standard errors at the occupation level. ***p<0.01, **p<0.05, *p<0.1.

### 1.2.2 Supplementary Table B2 — PSM: inverse-probability-weighted ATT estimates

| Measure | (1) Minor switch | (2) Intermediate switch | (3) Major switch |
| --- | --- | --- | --- |
| ATT — Total AI exposure (IPW) | 0.092*** | 0.083*** | 0.043* |
|  | (0.022) | (0.024) | (0.024) |
| ATT — Labor-saving AI exposure (IPW) | 0.097*** | 0.080*** | 0.032 |
|  | (0.021) | (0.024) | (0.023) |
| ATT — Labor-augmenting AI exposure (IPW) | 0.005 | 0.066*** | 0.028 |
|  | (0.020) | (0.019) | (0.021) |

*Notes*: Inverse-probability-weighted counterpart to nearest-neighbor PSM ATTs in main-text Table 3. Bootstrap standard errors (B = 200) in parentheses. Each treatment indicator equals one for workers whose relevant origin-occupation exposure is above its sample median; the propensity-score model uses the same 20 covariates as main-text Table 3. ***p<0.01, **p<0.05, *p<0.1.

### 1.2.3 Supplementary Table B3 — Industrial-robot IV: an independent automation measure

| Variable | (1) Minor switch | (2) Intermediate switch | (3) Major switch |
| --- | --- | --- | --- |
| ln(China robot density) | 0.075 | 0.108 | −0.095 |
|  | (0.049) | (0.052) | (0.087) |
| Wild bootstrap *p* (Webb) | 0.48 | 0.35 | 0.35 |
| First-stage partial Wald (KP-type) | 449.6 | 449.6 | 449.6 |
| First-stage partial R² | 0.888 | 0.888 | 0.888 |
| Observations | 2,446 | 2,446 | 2,446 |

*Notes*: 2SLS linear-probability estimates. Robot density is defined as $\log\left[ 1+\text{cumulative IFR robot stock}/\text{2010 industry employment}\times1,000 \right]$. Chinese robot density is instrumented with the analogous rest-of-world measure, following the identification logic of Acemoglu and Restrepo (2020). CLDS origin occupations are assigned to only five highly uneven International Federation of Robotics (IFR) industry groups. Standard errors are clustered at the industry-group level, and wild cluster bootstrap p-values use Webb weights with 9,999 replications. Neither inference procedure rejects zero at the 10% level for any transition distance. The first-stage statistics indicate instrument relevance but do not resolve the few-cluster limitation or establish the exclusion restriction. The exercise is exploratory and statistically inconclusive. Cross-referenced from main-text Section 4.2.

### 1.2.4 Supplementary Table B4 — Origin-destination exposure among switchers: total-exposure 2SLS estimates

| Variable | (1) Minor: destination R | (2) Minor: destination N | (3) Intermediate: destination R | (4) Intermediate: destination N | (5) Major: destination R | (6) Major: destination N |
| --- | --- | --- | --- | --- | --- | --- |
| Origin total AI exposure | 0.163*** | 0.084*** | 0.093*** | 0.062** | -0.140*** | -0.042 |
|  | (0.031) | (0.028) | (0.031) | (0.029) | (0.047) | (0.030) |
| R² | 0.220 | 0.126 | 0.190 | 0.111 | 0.123 | 0.067 |
| KP Wald F | 90.08 | 90.08 | 87.67 | 87.67 | 87.64 | 87.64 |
| Observations | 1,659 | 1,659 | 1,416 | 1,416 | 867 | 867 |

*Notes*: Total-exposure counterparts to main-text Table 6 for three nested samples of observed switchers. Outcomes are destination-occupation log labor-saving (R) and labor-augmenting (N) exposure. Origin total AI exposure is instrumented using the total-exposure instrument constructed in Eq. (3). Because origin total exposure predicts channel-specific destination outcomes, no unit-slope benchmark is applied. Stars test the null of zero. All specifications include individual and city controls and year and province fixed effects. Cluster-robust standard errors at the origin-occupation level are in parentheses. ***p<0.01, **p<0.05, *p<0.1.

### 1.2.5 Supplementary Table B5 — Within-worker exposure-change indicators among switchers: IV-LPM estimates

| Variable | (1) Minor: DownR | (2) Minor: UpN | (3) Intermediate: DownR | (4) Intermediate: UpN | (5) Major: DownR | (6) Major: UpN |
| --- | --- | --- | --- | --- | --- | --- |
| **Panel A: Decomposed (R + N)** | | | | | | |
| Origin labor-saving AI exposure | 0.237*** | −0.031 | 0.239*** | −0.019 | 0.251*** | −0.062** |
|  | (0.017) | (0.029) | (0.014) | (0.025) | (0.019) | (0.025) |
| Origin labor-augmenting AI exposure | 0.013 | −0.243*** | 0.016 | −0.261*** | 0.006 | −0.238*** |
|  | (0.025) | (0.030) | (0.022) | (0.028) | (0.030) | (0.026) |
| R² | 0.308 | 0.216 | 0.322 | 0.229 | 0.367 | 0.250 |
| KP Wald F | 26.67 | 26.67 | 25.24 | 25.24 | 28.31 | 28.31 |
| **Panel B: Total exposure** | | | | | | |
| Origin total AI exposure | 0.239*** | −0.139*** | 0.244*** | −0.137*** | 0.248*** | −0.170*** |
|  | (0.012) | (0.027) | (0.010) | (0.027) | (0.014) | (0.023) |
| R² | 0.296 | 0.108 | 0.311 | 0.099 | 0.354 | 0.151 |
| KP Wald F | 90.08 | 90.08 | 87.67 | 87.67 | 87.64 | 87.64 |
| Observations | 1,659 | 1,659 | 1,416 | 1,416 | 867 | 867 |

*Notes*: Selected-sample 2SLS linear-probability estimates for three nested samples of workers who switched at the minor-, intermediate-, and major-group margins. $\Delta R$ is destination-occupation labor-saving exposure minus origin-occupation labor-saving exposure, and $\Delta N$ is defined analogously for labor-augmenting exposure; all exposure measures are in log units. DownR = $1\{\Delta R<0\}$; UpN = $1\{\Delta N>0\}$. The estimates use three nested samples of observed switchers. Panel A jointly instruments origin R and N exposure; Panel B instruments origin total exposure, using the instruments constructed in Eq. (3). Because these indicators compare destination exposure with each worker’s own origin exposure, their coefficients are mechanically related to origin exposure levels. They are therefore interpreted descriptively rather than as tests of destination sorting, risk avoidance, or opportunity-seeking behavior. All specifications include individual and city controls and year and province fixed effects. Cluster-robust standard errors at the origin-occupation level are in parentheses. ***p<0.01, **p<0.05, *p<0.1.

### 1.2.6 Supplementary Table B6 — Descriptive exposure by initial total-exposure tertile (minor-group switching sample)

| Variable | Low — Mean | Low — SD | Middle — Mean | Middle — SD | High — Mean | High — SD | Full — Mean | Full — SD |
| --- | --- | --- | --- | --- | --- | --- | --- | --- |
| **Observations** | 486 |  | 607 |  | 566 |  | 1,659 |  |
| Origin total AI exposure | 0.329 | 0.327 | 1.711 | 0.456 | 3.089 | 0.568 | 1.776 | 1.192 |
| Origin labor-saving exposure | 0.218 | 0.234 | 1.515 | 0.623 | 2.910 | 0.648 | 1.611 | 1.204 |
| Origin labor-augmenting exposure | 0.159 | 0.171 | 0.435 | 0.417 | 1.076 | 0.864 | 0.573 | 0.686 |
| Destination labor-saving exposure | 1.190 | 1.051 | 1.589 | 1.113 | 2.044 | 1.195 | 1.627 | 1.174 |
| Destination labor-augmenting exposure | 0.478 | 0.552 | 0.616 | 0.713 | 0.856 | 0.768 | 0.657 | 0.707 |
| Years of education | 12.163 | 3.276 | 11.351 | 3.781 | 10.292 | 3.566 | 11.227 | 3.641 |

*Notes*: Entries are means and standard deviations for workers who switched at the minor-group margin. Tertiles are defined by origin-occupation total AI exposure. Origin exposure refers to the pre-switch occupation and destination exposure to the post-switch occupation; all exposure variables are in log units. The table is descriptive and should not be interpreted as a causal validation of the IV estimates. Cross-referenced from main-text Section 4.4.1.

### 1.2.7 Supplementary Table B7 — Post-transition job conditions among switchers: total-exposure 2SLS estimates

| Variable | Weekly hours | | | Skill-match satisfaction | | |
| --- | --- | --- | --- | --- | --- | --- |
|  | Minor | Inter. | Major | Minor | Inter. | Major |
| Origin total AI exposure | 0.390 | 0.471 | 0.408 | −0.012 | −0.016 | −0.019 |
|  | (0.391) | (0.405) | (0.575) | (0.015) | (0.016) | (0.018) |
| KP Wald F | 90.34 | 87.70 | 87.64 | 90.91 | 89.53 | 91.28 |
| Observations | 1,657 | 1,415 | 867 | 1,590 | 1,355 | 834 |

*Notes*: Selected-sample 2SLS estimates for three nested samples of workers who switched at the minor-, intermediate-, and major-group margins. The regressor is origin-occupation total AI exposure, instrumented using the total-exposure instrument constructed in Eq. (3). Weekly hours is continuous; skill-match satisfaction is a binary outcome estimated as a linear-probability model. KP Wald F denotes the Kleibergen–Paap weak-instrument F-statistic. All specifications include individual and city controls and year and province fixed effects. Cluster-robust standard errors at the origin-occupation level are reported in parentheses. Sample sizes vary across outcomes because of item non-response. Because the analysis conditions on observed switching, the estimates are interpreted as selected-sample relationships rather than as the full-sample LATE identified for switching incidence. Destination-job training-requirement estimates are reported separately in Supplementary Table B8. ***p<0.01, **p<0.05, *p<0.1.

### 1.2.8 Supplementary Table B8 — Destination-job training requirement among switchers: decomposed and total-exposure 2SLS estimates

| Variable | Minor | Inter. | Major |
| --- | --- | --- | --- |
| Panel A: Decomposed origin exposure | | | |
| Origin labor-saving AI exposure | −0.031** | −0.017 | −0.017 |
|  | (0.014) | (0.015) | (0.017) |
| Origin labor-augmenting AI exposure | 0.005 | −0.013 | −0.017 |
|  | (0.019) | (0.021) | (0.023) |
| KP Wald F | 26.67 | 25.24 | 28.31 |
| Observations | 1,659 | 1,416 | 867 |
| Panel B: Origin total exposure |  |  |  |
| Origin total AI exposure | -0.029** | -0.023* | -0.024* |
|  | (0.012) | (0.012) | (0.014) |
| KP Wald F | 90.08 | 87.67 | 87.64 |
| Observations | 1,659 | 1,416 | 867 |

*Notes*: Selected-sample 2SLS linear-probability estimates for whether the destination job requires training. The three switching samples are nested. The outcome is reported in the Supplementary Material because its welfare interpretation is ambiguous: a training requirement may reflect either a skill gap or entry into a more skill-intensive job. Panel A jointly instruments origin labor-saving and labor-augmenting exposure; Panel B instruments origin total exposure, using the instruments constructed in Eq. (3). All specifications include individual and city controls and year and province fixed effects. Cluster-robust standard errors at the origin-occupation level are in parentheses. ***p<0.01, **p<0.05, *p<0.1.

## 1.3 SI Section C — Search Keywords for Chinese AI Patent Retrieval

The Chinese AI patent corpus is retrieved from the patyee database using 93 AI-related keywords spanning AI hardware, core methods, and major application domains. Patents whose title or abstract contains at least one of these keywords are retained. The English translations of the 93 keywords are listed below; the original Chinese search terms are available in the replication package.

**Core methods**: Artificial Intelligence, Machine Learning, Deep Learning, Neural Networks, Reinforcement Learning, Supervised Learning, Fuzzy Logic, Classification Tree, Regression Tree, Support Vector Machine, Logic Programming, Pattern Recognition, Feature Extraction, Feature Recognition, Knowledge Representation and Reasoning, Knowledge Graph, Data Mining, Predictive Analytics, Expert System, Business Intelligence, Information Extraction, Intelligent Computing, Augmented Intelligence.

**Perception and recognition**: Computer Vision, Machine Vision, Image Recognition, Image Processing, Character Recognition, Optical Character Recognition (OCR), Text Recognition, Facial Recognition, Voiceprint Recognition, Palmprint Recognition, Gait Recognition, Iris Recognition, Vein Recognition, Fingerprint Recognition, Biometric Recognition, Identity Recognition, Emotion Recognition, Gesture Recognition, Speech Recognition, Semantic Recognition, Natural Language Processing, Speech Synthesis, Voice Interaction, Machine Translation, Question Answering System.

**Hardware and infrastructure**: AI Chip, Intelligent Chip, Intelligent Sensor, Wearable Product, Edge Computing, Cloud Computing, Internet of Things, Distributed Computing, Big Data Platform, Big Data Management, Big Data Analysis, Big Data Processing, Big Data Operations, Big Data Marketing, Big Data Risk Control.

**Application domains**: AI Product, Robot, Agent, Autonomous Driving, Assisted Driving, Driverless, Advanced Driver Assistance System (ADAS), Simultaneous Localization and Mapping (SLAM), Smart Home, Smart Speaker, Intelligent Voice, Augmented Reality, Virtual Reality, Human-Computer Interaction, Human-Computer Dialogue, Human-Machine Collaboration, Intelligent Customer Service, Smart Retail, Smart Healthcare, Smart Education, Smart Government Services, Smart Regulation, Smart Transportation, Smart Insurance, Robo-Advisor, Smart Banking, Smart Finance, Smart Agriculture, Smart Environmental Protection, Smart Elderly Care.

*Notes*: The full keyword list above contains 93 distinct keywords. Boolean OR is applied across the 93 keywords in patent retrieval. The retrieval result is the universe of disclosed Chinese AI-related patents from the patyee database for the period 2010–2018.
